# Bovine Lactoferrin and Lactoferrin-Derived Peptides Inhibit the Growth of *Vibrio cholerae* and Other *Vibrio* species

**DOI:** 10.3389/fmicb.2017.02633

**Published:** 2018-01-11

**Authors:** Erika Acosta-Smith, Karina Viveros-Jiménez, Adrian Canizalez-Román, Magda Reyes-Lopez, Jan G. M. Bolscher, Kamran Nazmi, Hector Flores-Villaseñor, Gerardo Alapizco-Castro, Mireya de la Garza, Jesús J. Martínez-Garcia, Jorge Velazquez-Roman, Nidia Leon-Sicairos

**Affiliations:** ^1^Programa Regional Para el Doctorado en Biotecnología, Facultad de Ciencias Químico-Biológicas, Universidad Autónoma de Sinaloa, Culiacán, Mexico; ^2^Centro de Investigación Aplicada para la Salud Pública (CIASaP), Facultad de Medicina, Universidad Autónoma de Sinaloa, Culiacán, Mexico; ^3^Hospital de la Mujer, Servicios de Salud de Sinaloa, Culiacán, Mexico; ^4^Departamento de Biología Celular, Centro de Investigación y de Estudios Avanzados del Instituto Politécnico Nacional, Mexico, Mexico; ^5^Department of Oral Biochemistry ACTA, University of Amsterdam and VU University, Amsterdam, Netherlands; ^6^Laboratorio Estatal de Salud Pública de Sinaloa, Culiacán, Mexico; ^7^Departamento de Investigación, Hospital Pediátrico de Sinaloa, Culiacán, Mexico

**Keywords:** lactoferrin, lactoferrin peptides, LFchimera, bactericide, *Vibrio cholerae*

## Abstract

*Vibrio* is a genus of Gram-negative bacteria, some of which can cause serious infectious diseases. Vibrio infections are associated with the consumption of contaminated food and classified in *Vibrio cholera* infections and non-cholera *Vibrio* infections. In the present study, we investigate whether bovine lactoferrin (bLF) and several synthetic peptides corresponding to bLF sequences, are able to inhibit the growth or have bactericidal effect against *V. cholerae* and other *Vibrio* species. The antibacterial activity of LF and LF-peptides was assessed by kinetics of growth or determination of colony forming unit in bacteria treated with the peptides and antibiotics. To get insight in the mode of action, the interaction between bLF and bLF-peptides (coupled to FITC) and *V. cholera* was evaluated. The damage of effector-induced bacterial membrane permeability was measured by inclusion of the fluorescent dye propidium iodide using flow cytometry, whereas the bacterial ultrastructural damage in bacteria treated was observed by transmission electron microscopy. The results showed that bLF and LFchimera inhibited the growth of the *V. cholerae* strains; LFchimera permeabilized the bacteria which membranes were seriously damaged. Assays with a multidrug-resistant strain of *Vibrio* species indicated that combination of sub-lethal doses of LFchimera with ampicillin or tetracycline strongly reduced the concentration of the antibiotics to reach 95% growth inhibition. Furthermore, LFchimera were effective to inhibit the *V. cholerae* counts and damage due to this bacterium in a model mice. These data suggest that LFchimera and bLF are potential candidates to combat the *V. cholerae* and other multidrug resistant *Vibrio* species.

## Introduction

The human innate-immune system is made up of a large variety of important components that attack or destroy any form of infection, in these components are included antibodies; white blood cells, antimicrobial proteins, and peptides, etc (Zaiou, 2007). This defense system is also found in other species of mammals, including bovines, sheep, and camels (Baveye et al., 1999). Although the development of new generation of antibiotics has rapidly progressed and gained popularity over the antimicrobial peptides, even the most powerful antibiotics have been unsuccessful to diminish morbidity and mortality due to the antimicrobial resistance showed by emergent multi-resistant strains of pathogens (Longworth, 2001; Spellberg et al., 2008). Antimicrobial peptides that are less prone to induction of resistance by bacteria are a class of substances that are now investigated to combat multi-drug resistant bacteria, with promising results (Ellison et al., 1990b; Garbacz et al., 2017; Greber and Dawgul, 2017). Such compounds include bovine lactoferrin (bLF) and LF-derived peptides (Ellison et al., 1990b). LF is an abundant iron-chelating protein present in colostrum and milk of most mammals, participating in the newborn protection against infections (Brock, 1980, 2002). LF is also present in mucosae and secreted bodily fluids such as bile, bronchioalveolar fluid, and intestinal and reproductive tract secretions, and it is produced and released by the polymorphonuclear neutrophils during inflammation (Brock, 2002). LF from bovine (bLF) and the LF derived peptides have been studied most extensively, due to exhibit antibacterial, antifungal, antiviral, and antiparasitic activities, in a direct way by a direct damage on pathogens and also by enhancing the mucosal immune function against pathogens (Brock, 2002; Orsi, 2004; Aguilar-Diaz et al., 2017; Juretic et al., 2017). bLF exerts its bactericidal action in two ways: indirectly, by limiting the amount of iron available for the growth and metabolism, and directly, by affecting the bacterial membrane (Ellison et al., 1988, 1990a; Ellison and Giehl, 1991; Orsi, 2004; Vogel, 2012). Other functions such as inhibition of bacterial adhesion or invasion to target cells, decrement of aggregation or biofilm development, have been also reported to LF and LF-peptides in bacteria (Singh et al., 2002; Orsi, 2004; Abbas et al., 2007; Juretic et al., 2017). The antimicrobial activity of LF is attributed to a region located at the N1-domain of the protein (Farnaud and Evans, 2003). In this sense, a peptide called lactoferricin B (LFcinB) is released from the N-terminus of bLF in during its passage through the intestine (Bellamy et al., 1992). Other antimicrobial peptides of the N1-domain have been identified and synthetically produced, for example, lactoferrampin (LFampin) (Van Der Kraan et al., 2004, 2005). Furthermore, a chimerical structure based in the active parts of the protein LF was designed and synthesized, this peptide contain the amino acids 17–30 of LFcinB and amino acids 265-284 of LFampin 265-284, the resulting peptides was called LF chimera (Bolscher et al., 2009). The bactericidal activity of LFchimera has been definitively stronger than that of the peptides (LFcin17-30 and LFampin265-284), as has been demonstrated in many experiments; due to lower concentrations, shorter incubation time, and salt concentrations present in the environment (needed for the growth of halophile bacteria) permit the bactericidal activity of LF chimera, compared with the peptides that conform this molecule which is not effective at these conditions (Bolscher et al., 2009; Haney et al., 2009; Leon-Sicairos et al., 2014). Otherwise, the microbicidal effect of LFchimera against *Candida* spp, *Vibrio parahaemolyticus, Staphylococcus aureus*, enterotoxigenic and enterohaemorragic *Escherichia coli*, or in the parasites *Entamoeba histolytica, Burkholderia thailandensis*, and *Leishmania pifanoi* has been established *in vitro* (Bolscher et al., 2009; Lopez-Soto et al., 2009, 2010; Flores-Villasenor et al., 2010, 2012; Kanthawong et al., 2014; Leon-Sicairos et al., 2014; Puknun et al., 2016).

Vibriosis is an infection caused by species of the *Vibrio* genus. *V. cholerae, V. parahaemolyticus*, and *V. vulnificus* are serious human pathogens (Thompson et al., 2004). *Vibrio cholerae* is the causative agent of cholera. It has been reported that the mortality rate of untreated cholera cases is about 50 to 60% (Frost, 1976; Faruque et al., 1998). Other *Vibrios* clinically significant for humans are *V. algynoyticus, V. parahaemolyticus*, and *V. vulnificus*. *V. alginolyticus* is medically important since it causes otitis and wound infection (Powell, 1999; Hernandez-Robles et al., 2016). The halophilic (salt-loving) *V. parahaemolyticus* has been identified as a leading cause of human gastroenteritis, associated to the consumption of raw or improperly cooked seafood (Ellison and Giehl, 1991; Su and Liu, 2007). Another halophilic *Vibrio* has recently been identified as *V. vulnificus*. This bacterium is an opportunistic pathogen that can cause infections of humans and other animals including fish. *V. vulnificus* is extremely harmful and is responsible for the devastating majority of reported seafood-related deceases in the United States (Warnock and Macmath, 1993; Powell, 1999; Jones and Oliver, 2009). The bacteria is located as a natural flora of coastal marine environments worldwide, for that it has been isolated from a seafood, shrimp, fish, oysters and clams, water, and sediments (Do Nascimento et al., 2001; Jones and Oliver, 2009; Jones et al., 2014).

It has been reported that the number of *V. cholerae* and *V. non-cholerae* cases has augmented increasingly in recent years. However; the major health problem is the emergence and spread of *V. cholerae* and *Vibrio non-cholera* strains antibiotics-resistant (Colwell, 1996; Faruque et al., 1998; Lipp et al., 2002; Sedas, 2007). For these reasons, the search for new compounds for Vibrio infections; treatment or prevention is needed. In previous work, it was demonstrated that LF had antibacterial effect against *V. cholerae* (Ellison and Giehl, 1991). Then; we reported that LF and the LFpeptides display antibacterial activity against a *V. parahaemolyticus* multidrug resistant strain, and also in *V. cholerae* O1 Inaba and non-O1 strains (Leon-Sicairos et al., 2009). In the present study; we continue the research of the bactericidal activity of bLF and bLF-derived peptides LFcin17-30, LFampin265-284 and LFchimera against *Vibrio* species resistant to antibiotics (including *V. cholerae* strains O1 and non-O1, *in vitro* and *in vivo*). In addition, we explore the mechanism of damage of these compounds into bacteria, and their synergism with antibiotics in the bactericidal effect.

## Materials and methods

### Lactoferrins, bacterial strains, and culture conditions

Bovine LF (bLF, 20% iron saturated) was kindly donated by Abial (Santander, Spain). The purity of bLF (>98%) was confirmed by SDS-PAGE gels using silver nitrate staining. LF concentration was measured by UV spectroscopy on the basis of an extinction coefficient of 15.1 (280 nm, 1% solution) (Valenti et al., 1999). The bLF iron saturation was about 20% as detected by optical spectroscopy at 468 nm on the basis of an extinction coefficient of 0.54 (100% iron saturation). LPS contamination of bLf, estimated by Limulus Amebocyte assay (LAL Pyrochrome kit, ThermoFicherScientific, Waltham, MA, USA), was equal to 0.7 ± 0.06 ng/mg of bLF. Synthetic peptides (LFcin17-30, LFampin265-284 and LFchimera) were obtained by solid phase peptide synthesis using Fmoc chemistry, as has been reported previously (Bolscher et al., 2009; Cutone et al., 2014).

The following *Vibrio* strains obtained by us were used: *V. cholerae* O1 Inaba, *V. cholerae* non-O1 (toxigenic), *V. fluvialis, V. alginolyticus, V. vulnificus*, and *V. furnissii* (Velazquez-Roman et al., 2012; De Jesus Hernandez-Diaz et al., 2015). Bacteria were incubated in Luria-Bertani medium (LB) (Difco, Becton Dickinson, USA) with 3% NaCl, incubated in agitation (5,000 rpm) and grown at 37°C for 16–18 h. In all the experiments in the presence of bLF or peptides, to avoid saturation with iron the ion was removed from the LB medium by incubation with Chelex-100 resin (5 g/l) in constant agitation at 4°C. After 16 h, the resin was taken off by filtration and finally the medium was sterilized (iron-depleted medium). The viability of bacterial cultures grown at these conditions was not affected. Additionally, all glass materials were treated with 6 M HCl to eliminate iron traces as previously reported (Leon-Sicairos et al., 2009).

### Growth inhibition in the presence of bLF and bLF-peptides

To determine the antibacterial activity of bLF and bLF peptides, ~1 × 10^7^ CFU/ml of *V. cholerae* O1 Inaba or *V. cholerae* non-O1 in 96-well microplates (Corning) containing 200 μl iron-depleted LB media were incubated at 37°C with 5, 10, 20, 30, and 40 μM bLF, LFcin17-30, LFampin265-284, or LFchimera, for 1, 2, 4, and 6 h. In parallel bacterial suspensions were treated with 25 μg/ml of Gentamicin or without additions as growth control. Bacterial growth was followed by measuring the OD660 nm of cultures. Next, the percentage of viable cells was estimated in relation to untreated cultures (without peptides or antibiotics). Viable cells were also counted as colony forming units/ml (CFU/ml) from serial 10-fold dilutions incubated in Muller-Hinton-Broth (MH broth), and then plated on MH agar plates at time and conditions afore mentioned. An electronic counter (CountTM, Heathrow Scientific) was used to count colonies. All experiments were repeated at least twice in triplicate. The synergistic effect of bLf on the antibiotic MIC was evaluated using the fractional inhibitory concentration (FIC). The interpretation of results was based on the following scale: FIC > 2 indicated a synergistic effect (Luna-Castro et al., 2014). All experiments were repeated at least twice in triplicate. Statistical significance was determined using a Student's *t*-test (*p* < 0.05), or ANOVA (with Bonferroni correction).

### Flow cytometry

To see if bLF and bLFpeptides cause membrane permeabilization, we used the staining with propidium iodide (PI), a fluorescent dye (this PI-assay is a quick assay that allowed us to compare the membrane damage by inclusion of PI, in a series of peptides under different conditions and incubation times). In brief, aliquots of 10^7^ CFU/ml of *V. cholerae* O1 and non-O1-strains were cultured in LB broth, harvested by centrifugation (5,000 rpm/5 min), washed three times with LB broth, and incubated with 40 μM bLF or 20 μM of either LFcin17-30, LFampin265-284, or LFchimera at 37°C for 2 h. Next, bacteria were washed and incubated with 10 mg/ml PI during 10 min at 4°C, washed five times with PBS (pH 7.4), and after fixed with 4% paraformaldehyde. Samples were washed twice with PBS and finally analyzed with a FACScan (Fluorescence-Associated Cell Scanner; Becton Dickinson, USA). Control experiments were carried out with bacteria either without the addition of bLF or bLFpeptides (membranes integrity control), or with 0.5% Triton X-100 (which permeabilizes bacterial membranes). All experiments were done at least twice in duplicate.

### Electron microscopy

*V. cholerae* O1 and non-O1 strains (10^8^ CFU/ml) cultures were incubated in iron-depleted LB without additions (negative control of damage), with 0.5% SDS (positive control of damage), or with 40 μM of bLF, or with 20 μM LFcin17-30, LFampin265-284 or 5 μM LFchimera, at 37°C for 1.5 h. Cells were collected, placed in tubes with PBS (pH 7.4) and fixed with 4% paraformaldehyde plus 0.5% glutaraldehyde. Next, samples were washed with distilled water and deposited on bare 200-mesh copper grids. Next, phosphotungstic acid (1%, pH 5.5, 30 s) was added, replicas were then dehydrated and finally studied with a transmission electron microscope (IEM2000Ex), operated at 100 kV.

### Confocal microscopy

The interaction of *V. cholerae* with bLF and the bLFpeptides was investigated by confocal microscopy. Briefly, 10^7^ CFU/ml of *V. cholerae* O1 cells were incubated in iron-depleted LB containing 2 μM FITC-labeled peptides for 30 min. Bacteria were centrifuged (5 min, 10,000 × g), resuspended and fixed (4% paraformaldehyde, pH 7.4 during 30 min at 37°C), washed twice and prepared to be examined under confocal microscopy. To find out whether bLF and bLFpeptides are recognized by the bacterial membrane of dead bacteria, *V. cholerae* O1 cells were fixed, then washed twice with PBS and incubated with 2 μM of FITC-bLF or FITC-labeled peptides for 30 min. After, samples were washed with PBS and mounted on slides and processed. All samples were analyzed under confocal microscopy by using a confocal laser-scanning microscope (Leica, Heidelberg, Germany).

### Effect of bLF and lfchimera on the antibacterial activity of classic antibiotics used against *vibrio* spp.

*V. cholerae* O1 Inaba, *V. cholerae* non-O1, *V. vulnificus* (resistant to tetracycline and ampicillin), *V. fluvialis* (resistant to ampicillin and cefotaxime), *V. alginolyticus* (resistant to ampicillin and tetracycline), and *V. furnissii* (resistant to ampicillin), were used to determine whether bLF and LFchimera potentialize the bactericidal activity of common antibiotics. First, to test the resistance level to common antibiotics, the bacterial strains were grown with or without gentamicin (2–25 μg/ml), tetracycline (2.5–20 μg/ml), chloramphenicol (2.5–30 μg/ml), or ampicillin (2.5–32 μg/ml); bactericidal activity of LFchimera (1, 5, 10, and 20 μM) was tested in parallel. Next the antibiotics ampicillin (2.5–32 μg/ml), chloramphenicol (2.5–30 μg/ml), and tetracycline (2.5–20 μg/ml) were tested in the presence of a sub-MIC concentration of bLF (10 μM) or LFchimera (1 μM). Percentage of viable cells was determined in relation to cultures without added peptides or antibiotics. All experiments were repeated at least twice in triplicate.

### *In vivo* model

#### Bacterial strain and culture conditions

The *Vibrio cholerae* O1 serotype Inaba was maintained in TCBS agar (BD, USA) at 37°C during 24 h. Bacterial cultures (used for mice inoculations) were routinely grown on LB agar plates with 100 mg/ml streptomycin for 18 h and finally were grown with shaking in LB broth with antibiotic at 37°C to mid-log phase. The OD620 nm was adjusted to 1 and this inoculum was used in the assays.

#### Inoculation of *vibrio cholerae* O1 serotype inaba in mice and treatments

**Six** to eight-week-old female BALB/cAnNHsd mice (Harlan Laboratories, Inc., Mexico), were purchased and housed under specific-pathogen-free conditions as stipulated by the Ethical Committee for Laboratory Animals in Faculty of Medicine of UAS and were divided into five groups. Mice were given 0.1% (w/v) Streptomycin for 3 days to ablate normal flora. A day prior to inoculation, food was removed from cages to empty the stomach. Mice were injected intraperitoneally with 12.5 mg/kg xylazine. When mice were deeply sedated, 50 μl of 0.5 M NaHCO3 was administered intragastrically immediately followed by 500 μl of bacterial suspension (2.5 × 10^7^ CFU). After inoculation, mice were kept with free access to food and sterile water without streptomycin. Then, after 4 h post-inoculation (after infection and symptoms were established) different treatments were administered into mice each 12 h for 3 days. Treatment doses administered were as follows; 65 mg/Kg of bLF, 5 μg/Kg of LFchimera, and 14 mg/Kg of Tetracycline (Sigma Inc. USA). Mice of the control group were administered 0.5 ml of PBS instead of antimicrobial agents. All of the mice were housed in groups consisting of 10 mice each and permitted food and water *ad libitum*.

#### Identification of *vibrio cholerae* O1 in infected mice

In order to evaluate the *V. cholerae* mice infection procedure and establishment, a disposable 1 μl plastic inoculation loop (diameter 2.0 mm) was introduced into the rectum. The loop was turned around to obtain *V. cholerae* O1 from the inner surface of the rectum. The tip of the loop was subsequently clipped into a 1.5 ml tube containing 500 μl of enriched alkaline peptone water and incubated for 18 h at 37°C. The bacteria from slopes were streaked onto TCBS agar and CHROMagarTM Vibrio to confirm the infection. Once the infection was demonstrated (after 4 h) the treatments were administered.

#### CFU enumeration of *vibrio cholerae* O1 in feces and intestines of mice

Briefly, fresh feces of mice were weighed and suspended in PBS. Then, samples were homogenized and serial dilutions were prepared and plated onto LB agar plates with 100 mg/ml streptomycin for 24 h at 37°C. For confirmation, developed colonies were counted and then plated onto CHROMagarTM Vibrio (CHROMagar; Paris, France). On the other hand, the intestines were dissected, homogenized in PBS and serial dilutions were prepared in PBS and plated in LB agar plates with 100 mg/ml streptomycin. Finally, colonies were counted and plated onto CHROMagarTM Vibrio.

## Results

### bLF and bLFpeptides inhibited the growth of *V. cholerae* O1 and non-O1 strains

**Figure 1:**
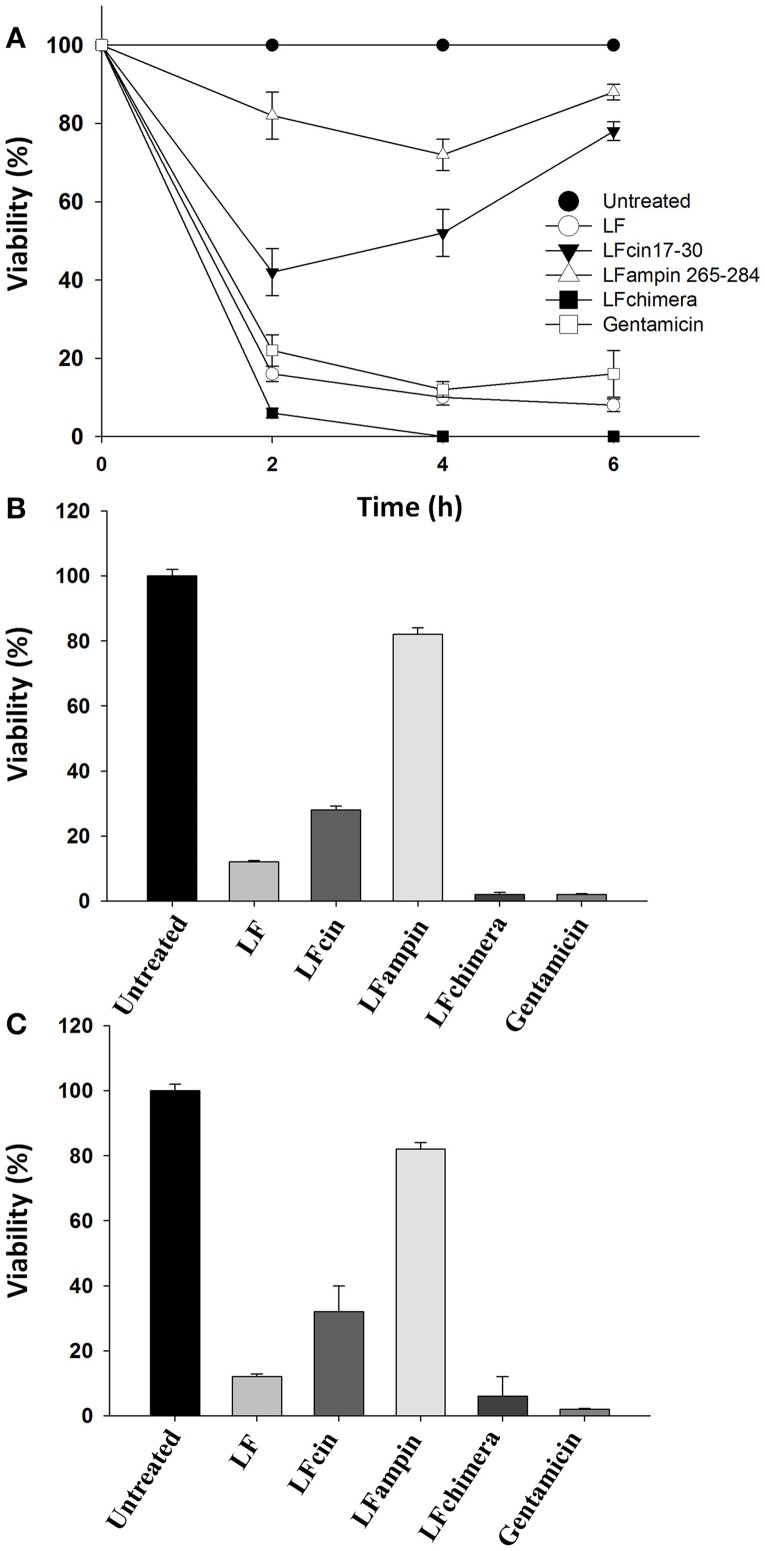
Bactericidal effect of bLF and LF-peptides on *Vibrio cholerae* O1 and non-O1. Approximately 1 × 10^7^ CFU/ml of *V. cholerae* O1 and non-O1 strains were incubated with bLF and LFpeptides solutions at final concentrations of 40 μM bLF or 20 μM of LFcin17-30, LFampin265-284, respectively; and 5 μM of LFchimera at 37°C with constant agitation for 1, 2, 4, or 6 h. Bacteria grown in LB broth were used as a control for optimal growth and 100 μM of Gentamicin was used as a control for growth inhibition. Bacterial growth was followed by measuring the OD660 nm of cultures. Percentage of viable cells was determined in relation to cultures Gentamicin without peptides or antibiotics **(A)**. All Experiments were repeated at least twice in triplicates. *V. cholerae* O1 **(B)** and non-O1 **(C)** strains were incubated with bLF and LFpeptides solutions at final concentrations of 40 μM bLF and 20 μM LFcin17-30, LFampin265-284, and 5 μM LFchimera; respectively. Viability was monitored by enumerating colony forming units CFU/ml (viable cells) obtained from serial 10-fold dilutions plated onto MH agar **(B,C)**. Percentage of viable cells was calculated relative to viable bacteria untreated grown in MH agar. Experiments were performed in triplicate; mean and standard deviation are indicated. Statistical significance was determined using a Student's *t*-test for *P*-values < 0.05, and ANOVA (with Bonferroni correction).

Historically, *V. cholerae* O1 and non-O1 strains have caused more problems to human health that other *Vibrio* species. So, we used these strains in order to test the antibacterial activity of bLF and LFpeptides. The ability of bLF and LFpeptides (LFcin17-30, LFampin265-284, and LFchimera) to inhibit the growth of *V. cholerae* O1 and non-O1 strains of *V. cholerae* was analyzed by measuring the growth in untreated and treated cells after several incubation times. Results shown that LFchimera had the best bactericidal effect, since 5 μM inhibited the growth until 6% at 2 h of incubation (with respect to the untreated bacteria). This inhibition was better than those exerted by 25 μg/ml of Gentamicin which inhibited the culture until 22% during the first 2 h of incubation. Percentage of growth inhibition in cultures treated with 40 μM bLF was 16, 42% with 20 μM LFcin17-30 and 82% with LFampin265-284 (Figure 1A). In addition, only LFchimera at a concentration of 5 μM showed bactericidal activity after 4 and 6 h incubation. Gentamicin at 25 μg/ml showed low percentage of viability without any increase within 6 h, whereas in the presence of peptides LFcin17-30 and LFampin265-284 a decrease in viability was only found after 2 h and followed by a recovery of the viability after 4 and 6 h (Figure 1A).

On the other hand, by CFU counts after 2 h of incubation, *V. cholerae* O1 cultures treated with 5 μM LFchimera and 40 μM bLF were significantly reduced (until 2 and 6% respectively, relative to untreated bacteria) and the effect were similar to the bactericidal activity of Gentamicin (6% growth inhibition relative to untreated bacteria) (Figure 1B). However, in *V. cholerae* non-O1 strain Gentamicin and LFchimera apparently inhibited the cultures with the same efficacy (Figure 1C). In all treatments and incubation times (longer than 2 h) LFchimera completely inhibited the growth of *V. cholerae* O1 and *V. cholerae* non-O1 strains (Figures 1B, C; respectively). The bactericidal activity of LFchimera was stronger than those of bLF, LFcin17-30, and LFampin265-284 (6, 22, and 82% of growth inhibition, relative to untreated bacteria).

### Lactoferrin and lactoferrin-derived peptides showed combined effect with antibiotics and inhibited the growth of *vibrio*

*V. cholerae* O1 Inaba, *V. cholerae* non-O1, *V. vulnificus* (resistant to tetracycline and ampicillin), *V. fluvialis* (resistant to ampicillin and cefotaxime), *V. alginolyticus* (resistant to amplicillin and tetracycline), and *V. furnissii* (resistant to ampicillin) were used to determine whether bLF and LFchimera, each in combination with common antibiotics increase the bactericidal effect.

In the results, the combination of 1 μM LFchimera plus 2.5 μg/ml ampicillin were able to inhibit more than 95% of growth of *V. vulnificus, V. fluvialis, V. alginolyticus*, and *V. furnissii*. Similar effects on growth inhibition (more than 95%) were found with concentrations of 5 μM LFchimera (Table 1), or more than 32 μg/ml ampicillin, suggesting that the combination of LFchimera and ampicillin (1 and 2.5 μg/ml) can inhibit the growth of *Vibrio* spp. resistant to ampicillin (Table 1). On the other hand, by using a combination of LFchimera with tetracycline, the combination of 1 μM LFchimera plus 2.5 μg/ml of tetracycline was able to inhibit more than 95% of the growth of *V. vulnificus*; this inhibition growth is only reached with concentrations of 5 μM of LFchimera or more than 20 μg/ml of tetracycline (Table 1). A mixture of 1 μM LFchimera and 2.5 μg/ml chloramphenicol inhibited more than 95% of the growth of *V. cholerae* O1 and non-O1 strains, whereas this growth inhibition level was only reached by using concentrations of 5 μM LFchimera or 30 μg/ml chloramphenicol (Table 1). Ten microliters bLF also had synergism or combined effect with antibiotics in the strains above mentioned, whereas without antibiotics the concentration needed to inhibit more than 95% was 10 μM bLF. These data suggest that LFchimera and bLF combined with low concentrations of antibiotics have bactericidal effect in multidrug resistant strains of genus *Vibrio* (Table 1). According to the calculation of FICs, both bLF and LFchimera have synergistic effects when they were used with the antibiotics, suggestion that they could improve the management of *Vibrio* spp multidrug resistant strains to antibiotics *in vivo*.

**Table 1:** Effects of LFchimera and bLF combined with antibiotics on *Vibrio* spp growth.

**Concentrations (**μ**g/ml) of antibiotics for causing more than 95% growth inhibition**			**Concentrations (**μ**g/ml) of antibiotics for causing more than 95% growth inhibition**		
**Strain**	**Ampicillin without LFchimera**	**Ampicillin with 1 μM LFchimera**	**Strain**	**Ampicillin without bLF**	**Ampicillin with 10 μM bLF**
*V. vulnificus*	32	2.5	*V. vulnificus*	32	2.5
*V. fluvialis*	32	2.5	*V. fluvialis*	32	2.5
*V. alginolyticus*	32	2.5	*V. alginolyticus*	32	2.5
*V. furnissii*	32	2.5	*V. furnissii*	32	2.5
**Strain**	**Tetracycline without LFChimera**	**Tetracycline with 1** μ**M LFChimera**	**Strain**	**Tetracycline without LF**	**Tetracycline with 10** μ**M LF**
*V. vulnificus*	20	2.5	*V. vulnificus*	20	2.5
**Strain**	**Cloramphenicol without LFChimera**	**Cloramphenicol with 1** μ**M LFChimera**	**Strain**	**Cloramphenicol without LF**	**Cloramphenicol with 10** μ**M LF**
*V. cholerae O1*	30	2.5	*V. cholerae O1*	20	2.5
*V. cholerae non O1*	30	2.5	*V. cholerae non O1*	20	2.5

*An inoculum (OD 0.005 at 660 nm) was used in each experiment. Viability relative to the 100% growth in untreated bacteria cultures was determined by measuring the OD every 30 min during 1.5 h. Data are mean values of two experiments performed in triplicate. Standard deviations were less that 6.2% in each experiment*.

### bLF and bLFpeptides cause damage on *vibrio cholerae* O1 and non-O1 strains

**Figure 2:**
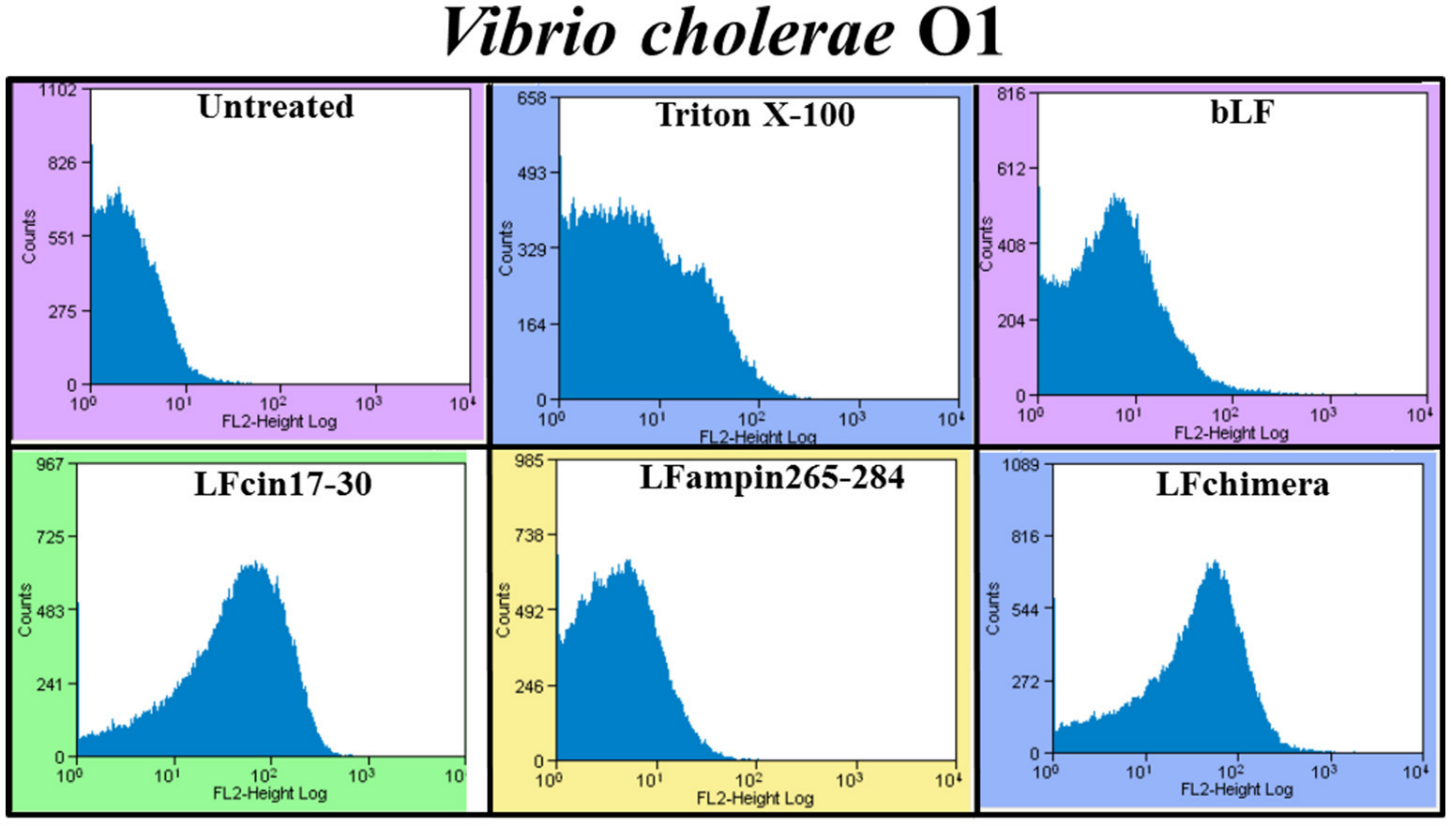
Determination of membrane permeabilization in *Vibrio cholerae* O1 treated with bLF and LF peptides. *V. cholerae* O1 was incubated with 40 μM LF, or with 20 μM LFcin17-30, or LFampin265-284, respectively; or 5 μM LFchimera, at 37°C with constant agitation for 2 h. Then, samples were processed and stained with the fluorescent dye Propidium iodide. Untreated bacteria were used as control of membrane integrity and 0.5% Triton X-100 treated bacteria were used as control of permeabilized membranes. Experiments were performed at least twice in duplicate. Samples were processed to be analyzed by Flow Cytometry.

The effect of bLF and bLFpeptides on the bacterial membrane integrity was investigated using the fluorescent dye PI (it only enters in permeabilized cells). PI was measured under flow cytometry. After the treatment, almost all *V. cholera* cells had taken up the dye fluorescence upon incubation with 5 μM LFchimera, 20 μM bLF LFcin17-30, and LFampin265-284; indicating that the bacterial membrane was permeabilized by the peptides (Figure 2). The treatment with Triton X-100 used as a positive control of bacterial permeabilization also stained bacterial cells, corroborating that this treatment damaged the bacteria (Figure 2).

**Figure 3:**
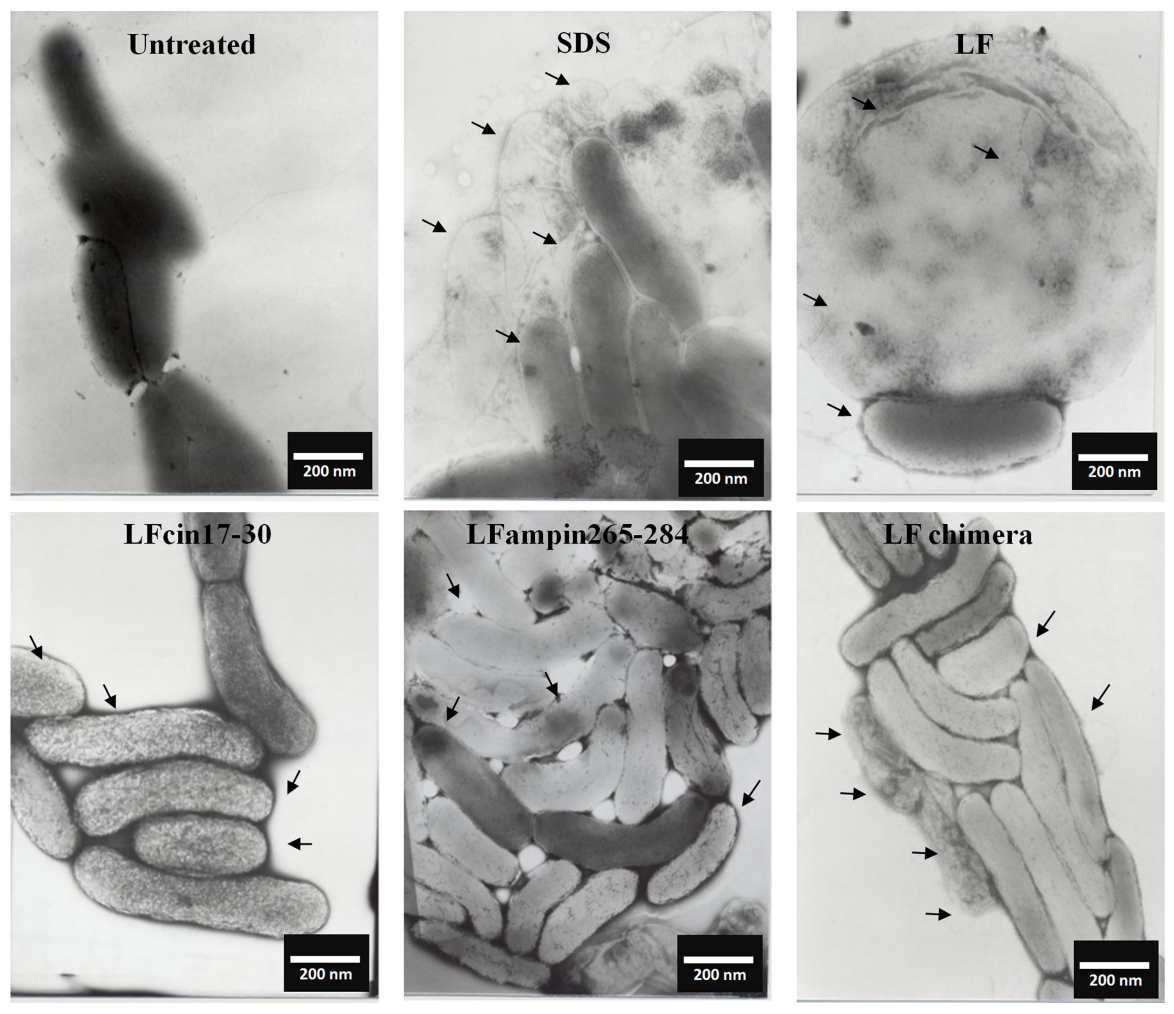
LF and LFpeptides cause ultrastructural damage to *Vibrio cholerae* O1 cells. *V. cholerae* O1 cells (1.0 × 10^8^ cells/ml) were incubated in LB alone (negative control for damage) or with 0.5% SDS (positive control for damage), or with 40 μM of bLF, 20 μM LFcin 17-30 or LFampin265-284 respectively, or 5 μM LFchimera for 1.5 h at 37°C. Cells were harvested, resuspended in PBS and fixed with 4% *para*-formaldehyde plus 0.5% glutaraldehyde. Next, bacterial samples were placed on 200-mesh Formvar-coated copper grids (3%), post-stained with phosphotungstic acid and examined with a JEOL electron microscope JEM1400 at 40 kv.

**Figure 4:**
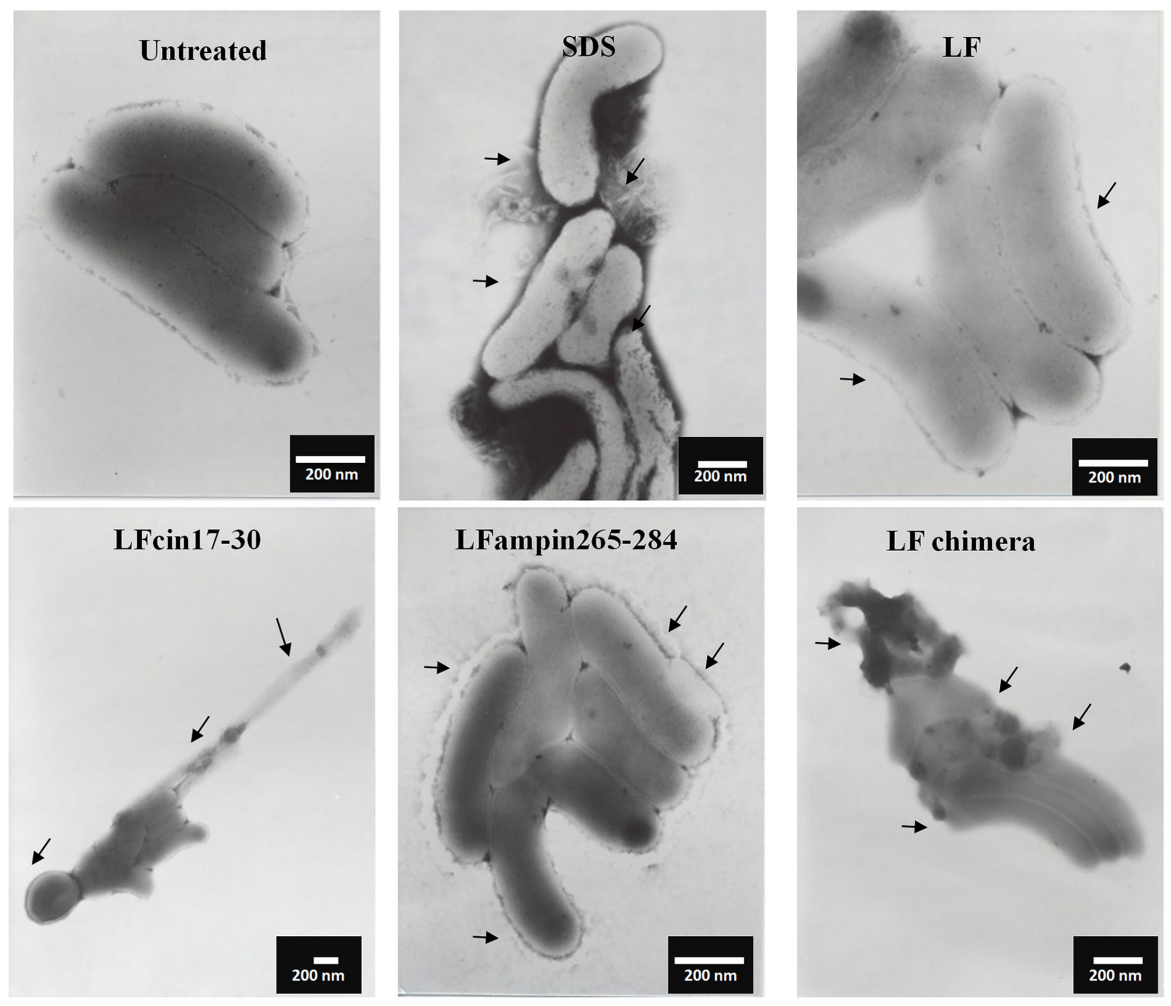
LF and LFpeptides cause ultrastructural damage to *Vibrio cholerae* non-O1 cells. *V. cholerae* non-O1 cells (1.0 × 10^8^ cells/ml) were incubated in LB alone (negative control for damage) or with 0.5% SDS (positive control for damage) or with 40 μM of bLF, or 20 μM LFcin 17-30 and LFampin265-284 respectively, or 5 μM LFchimera, for 1.5 h at 37°C. Cells were harvested, resuspended in PBS and fixed with 4% *para*-formaldehyde plus 0.5% glutaraldehyde. Next, bacterial samples were placed on 200-mesh Formvar-coated copper grids (3%), post-stained with phosphotungstic acid and examined with a JEOL electron microscope JEM1400 at 40 kv.

Similar incubations of *V. cholerae* O1 analyzed by SEM after negative staining showed severe membrane damage such as vesicularization, the occurrence of protrusions and filamentation (Figure 3, arrows). The same damage was found in *V. cholerae* non-O1 cells treated with bLF and LFpeptides (Figure 4). These results demonstrate that LFchimera and peptides destabilize the bacterial membrane integrity and also that LFchimera has a higher activity than bLF and LF peptides.

### bLF and bLFpeptides interact with *vibrio cholerae* strains

**Figure 5:**
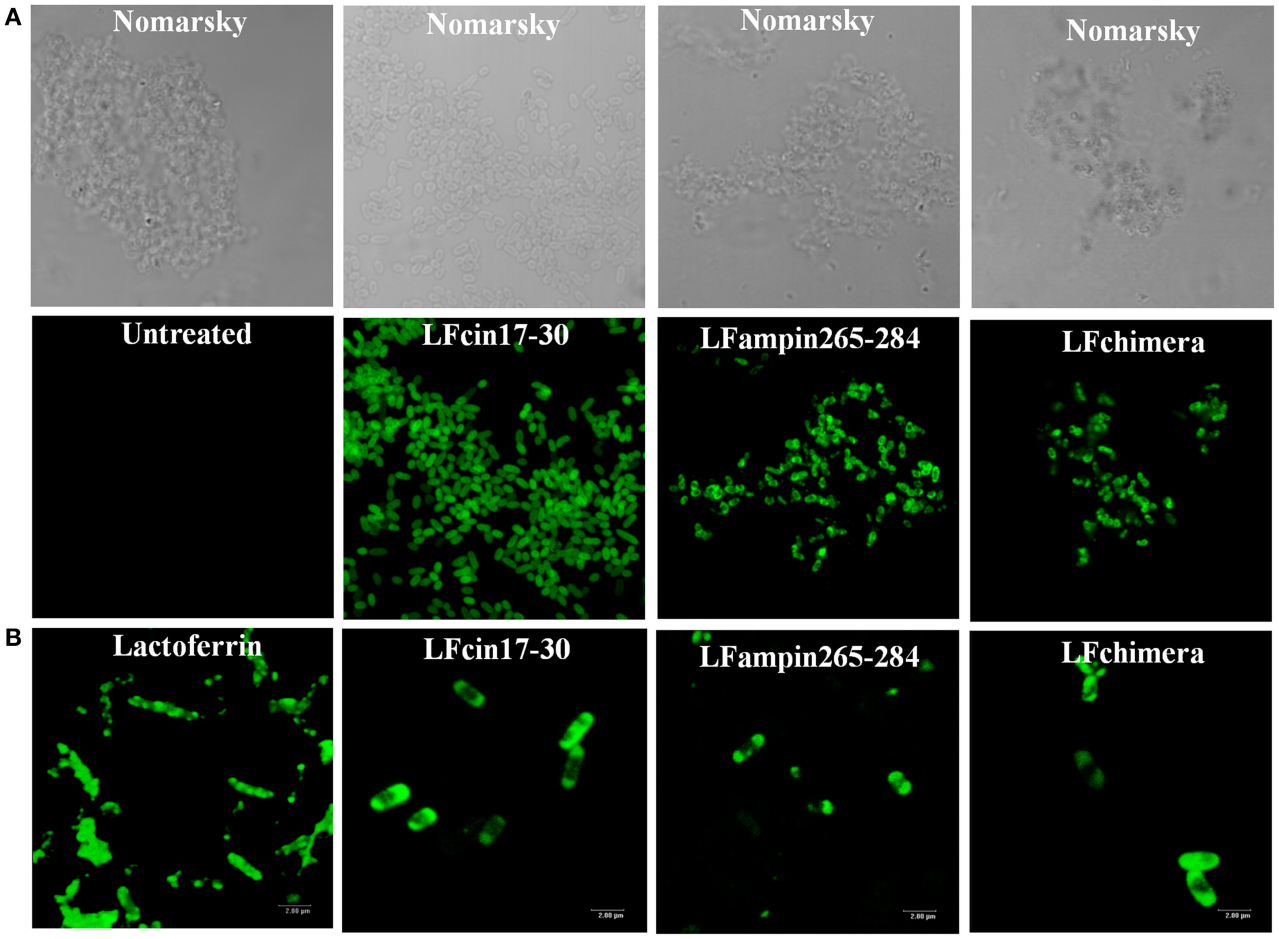
Interaction of Lactoferrin derived peptides with *Vibrio cholerae* O1 and non-O1 strains. *Vibrio cholerae* O1 cells (10^7^ CFU/ml) were incubated with 2 μM FITC-labeled peptides for 30 min. Bacteria were centrifuged (5 min, 10,000 × g), resuspended and incubated with 2 μM of FITC-bLF or FITC-labeled peptides for 30 min **(A)**, or fixed (4% paraformaldehyde, pH 7.4 during 30 min at 37°C) **(B)**, washed and then incubated with 2 μM bLF and LFpeptides as before was described. In both cases samples were washed twice with PBS mounted on slides and processed. All samples were analyzed under confocal microscopy by using a confocal laser-scanning microscope (Leica, Heidelberg, Germany). Bar 20 nm.

The interaction of bLF and LFpeptides was investigated by confocal microscopy. In the results, we observed that the peptides LFcin17-30, LFampin265-284, and LFchimera interact with *V. cholerae* bacteria (Figure 5A). On the other hand, in fixed bacteria the fluorescent compounds were found interacting with the bacteria, indicating that the membrane of *V. cholerae* contains components which are recognized by the peptides (Figure 5B).

### Bovine lactoferrin and lactoferrin chimera reduce damage on intestine and cecum of mice infected with *vibrio cholerae*

**Figure 6:**
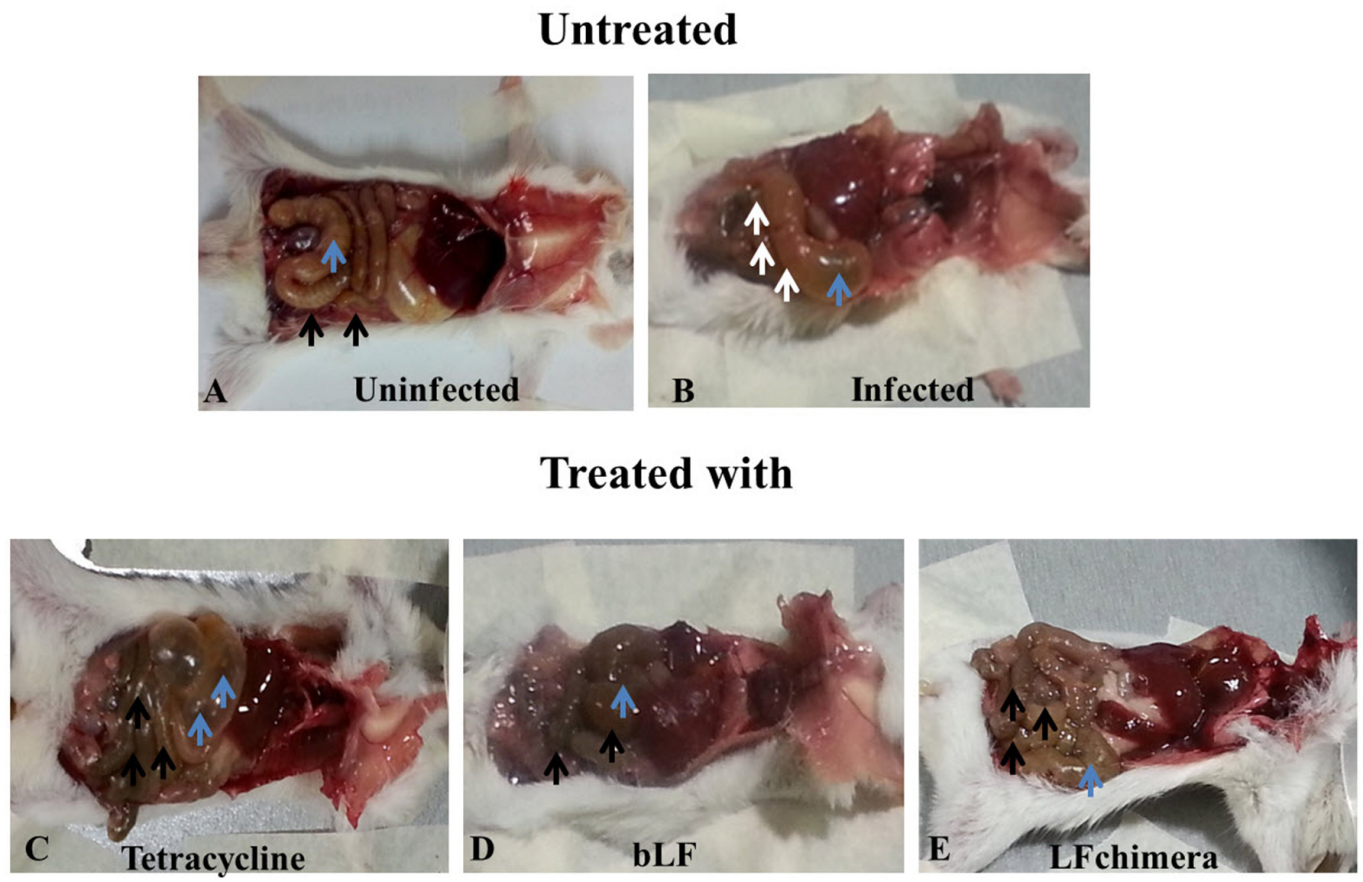
Alterations in gut morphology of mice. Representative macroscopic images for the gross morphological alterations of the small intestine and caecum from mice at 24 h of treatment. **(A)** Small intestine Black arrows indicate normal small intestine in the uninfected group (blue arrowhead) indicate normal caecum. **(B)** White arrows point to injury in small intestine in the infected and untreated group (blue arrow) shows caecum is swelling. **(C)** Black arrows indicate injury in small intestine (blue arrows) caecum is swelling and enlarged in the tetracycline group. **(D)** Black arrows indicate normal small intestine as in A (blue arrowhead) indicates normal caecum. **(E)** Black arrows indicate normal small intestine in the LFchimera group as in D (blue arrow) indicate caecum.

Mice infected with *V. cholerae* developed symptoms such as diarrhea, weakness, and abdominal tremor after 4 h of infection. Additionally; the infection was confirmed by counting *V. cholerae* obtained from rectal swabs (data nor shown), Once infection was confirmed, mice were treated with bLF, LFchimera, and tetracycline. Twenty-four post-infection three mice were sacrificed in order to see the effectiveness of the treatments. In the results, representative macroscopic images are shown for the gross morphological alterations of the small intestine and caecum from mice at 24 h of treatment (Figure 6). In a mouse of the uninfected group black arrows indicate the typical appearance of a normal small intestine, and blue arrowheads indicate normal caecum (Figure 6A). In a mouse from the infected and untreated group, white arrows point to injury in small intestine and blue arrow shows caecum is swelling (Figure 6B). A mouse from the group treated with tetracycline black arrows indicate injury in small intestine and blue arrows shows caecum is swelling and enlarged (Figure 6C). Interestingly, a mouse from the group treated with bLF Black arrows indicate normal small intestine as in Figure 6A and blue arrows indicates normal caecum (similar to macroscopic findings of uninfected mice). A mouse treated with LFchimera shows black arrows indicating a normal small intestine and blue arrow indicates a normal caecum. Similar results were observed in other mice sacrificed. These results indicated that bLF and LFchimera have the capacity to diminishing macroscopic damage induced by *V. cholerae* in intestines and cecum in Mice.

### Bovine lactoferrin and lactoferrin chimera reduce *vibrio cholerae* counts in feces and intestines

**Figure 7:**
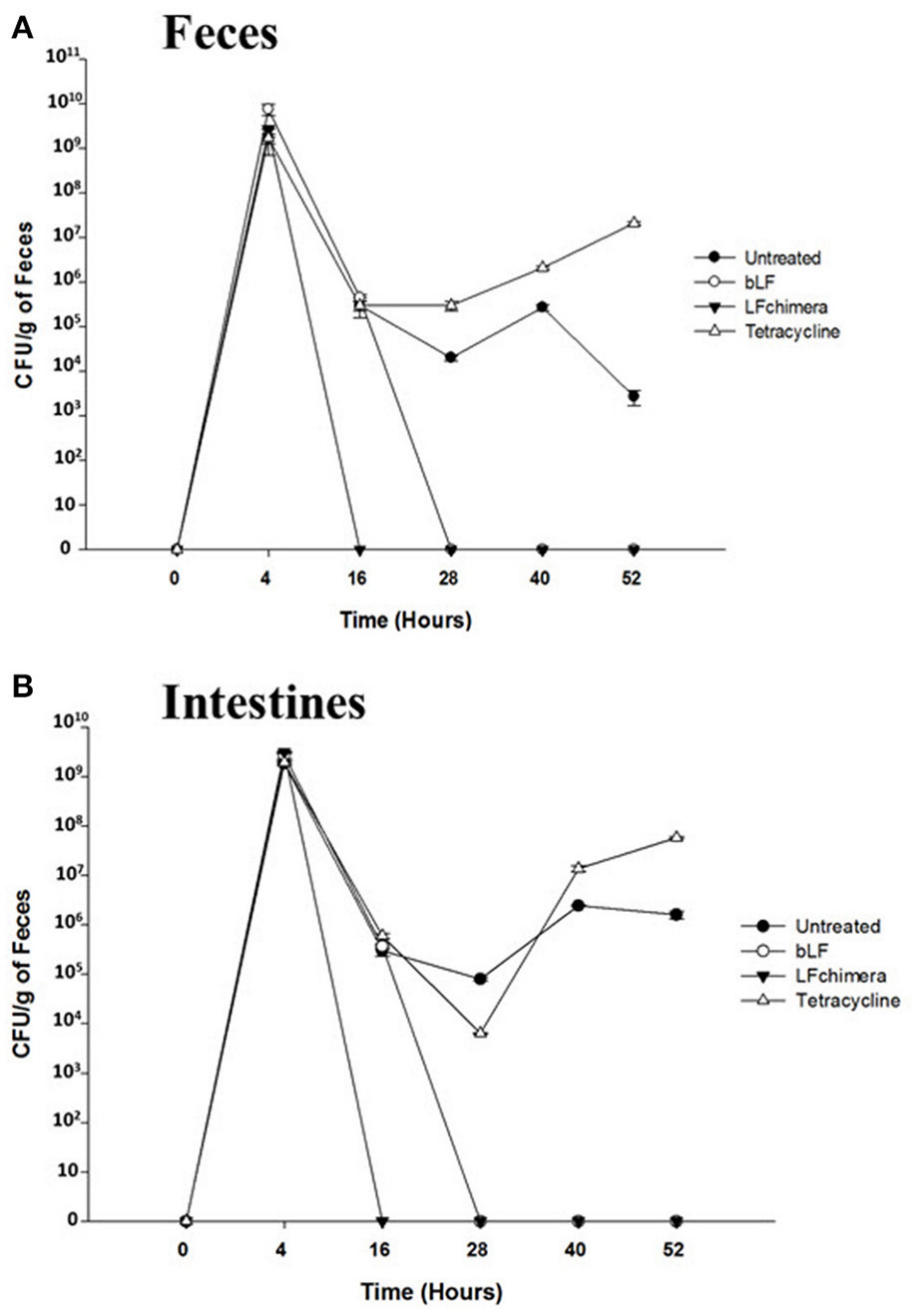
LF chimera and bLF diminish *Vibrio cholerae* O1 counts in **(A)** feces and **(B)** intestine. Recovery feces and intestines from mice infected and treated were homogenized and serial dilutions were prepared and plated onto LB agar plates with 100 mg/ml streptomycin for 24 h at 37°C. For confirmation, developed colonies were counted and then plated onto CHROMagar™ *Vibrio* (CHROMagar; Paris, France). On the other hand, the intestines were dissected, homogenized in PBS and serial dilutions were prepared in PBS and plated in LB agar plates with 100 mg/ml streptomycin. Finally, colonies were counted and plated onto CHROMagar™ *Vibrio*.

*V. cholerae* counts diminished in feces (Figure 7A) and intestines (Figure 7B) from mice infected and treated with bLF and LFchimera after the first dose administered (Figure 7), compared with infected and untreated animals (Positive control of infection). In the group treated with LFchimera, *V. cholerae* was undetected in feces and intestines after 12 h of treatment, in the group treated with bLF *V. cholerae* was undetected until 24 h of treatment and with tetracycline the minimal bacterial count was done during 12–16 h of treatment, and then the bacteria recovered its growth in feces and intestines. The results show that LFchimera and bLF kill *V. cholerae* in *in vivo* model.

## Discussion

Antimicrobial resistance is a global health concern because the infections can be more severe and difficult to treat (Bonomo, 2000). This is a consequence in part by overuse and misuse of antibiotics and represents a serious health concern throughout the world (Longworth, 2001). In this sense, in this problem is included the development of antibiotic resistance by *Vibrio* species (Rahmani et al., 2012; Sperling et al., 2015). The genus *Vibrio* includes at least 12 species pathogenic to humans. In these species are included *V. cholerae, V. parahaemolyticus, V. vulnificus, V. alginolyticus, V. furnissii, V. fluvialis, V. damsela, V. hollisae, V. metschnikovii*, and *V. mimicus* (Altekruse et al., 2000). Pathogenic *Vibrio* can cause both intestinal and extra-intestinal illnesses. *Vibrio* infections potentially requiring antimicrobial therapy fall into three distinct syndromes; (1) cholera; caused by either *V. cholerae* O1 and other serogroups such as non-o1 or other species (*V. parahaemolyticus*), (2) Soft tissue infections; due to *V. vulnificus*, and (3) sepsis due to *V. vulnificus* and other *Vibrio* (Powell, 1999). Acquired multidrug resistant in *V. cholerae* O1 and other pathogenic *Vibrio* is now common and firmly established wherever infections occur (Dhar et al., 1996; Elmahdi et al., 2016).

In this work, we demonstrated that bLF and LFchimera have bactericidal activity against *V. cholerae* O1 Inaba, *V. cholerae* non-O1 (toxigenic), *V. vulnificus* (resistant to tetracycline and ampicillin), *V. fluvialis* (resistant to ampicillin and cefotaxime), *V. alginolyticus* (resistant to amplicillin and tetracycline), and *V. furnissii* (resistant to ampicillin). Previous works have reported the bactericidal activity of bLF in *V. cholerae* (Arnold et al., 1980; Ellison and Giehl, 1991); however, the mechanism of action or damage was not investigated in detail. In regards with the bactericidal activity of bLF and LFpeptides on *V. cholerae*, the peptide LFchimera and the protein bLF had the best bactericidal activity (Figure 1). LFchimera was more effective to inhibit the growth of *V. cholerae* strains and other *Vibrio* spp., compared with its peptides of origin (LFcin17-30 and LFampin265-284). *V. cholerae* strains were also susceptible to both peptides; however, this antibacterial activity remained curiously lower when was compared to the antibacterial action of Gentamicin (drug used as a negative control for growth), or when was compared with the action of bLF and LFchimera (Figure 1). Nonetheless, LFcin17-30 and LFampin265-284 may be antibacterial, due they were able to damage membranes and cause disruption on *V. cholerae* cells (Figures 2–4). The more effective antibacterial ability of LFchimera compared to the effect reached with native bLF, LFcin17-30, and LFampin265-284 peptides has been reported for other bacteria, as well as parasites or fungi (Bolscher et al., 2009; Kanthawong et al., 2014; Leon-Sicairos et al., 2014). These differences on the effect could be due to the LFchimera structure (Haney et al., 2012a,b). An obvious question is why human or bovine LF doesn't prevent the infection by *Vibrio* species? We speculate that human LF present in mucosae and fluids, or released by neutrophils, or bLF ingested from dairy milk products is not enough to combat *Vibrio* spp infections.

As we found that LFchimera and bLF had the best bactericidal activity, we investigate the effects of them combined with low concentrations of antibiotics on multidrug resistant *Vibrio* strains. In the results, apparently a combined effect was found when antibiotics were mixed with bLF and LFchimera (Table 1). It is interesting that LFchimera mixed with ampicillin inhibited the growth of *V. vulnificus, V. fluvialis, V. alginolyticus*, and *V. furnissii* and also the growth of some *Vibrio* spp resistant to ampicillin (Table 1). On the other hand, the combination of LFchimera tetracycline inhibited the growth of *V. vulnificus* (Table 1). A mixture of LFchimera and chloramphenicol inhibited the growth of *V. cholerae* O1 and non-O1 strains, whereas this growth inhibition level was only reached by using higher concentrations of LFchimera or chloramphenicol (Table 1). bLF also had synergism or combined effect with antibiotics in the strains above mentioned, whereas without antibiotics the concentration needed to inhibit more than 95% was higher. These data suggest that LFchimera and bLF combined with low concentrations of antibiotics have bactericidal effect in multidrug resistant strains of genus *Vibrio* (Table 1). According to the calculation of FICs, both bLF and LFchimera had synergistic effects when they were used with the antibiotics, suggesting that they could improve the management of *Vibrio* spp multidrug resistant strains *in vivo*. In regards with this data, we speculate that the magnified effect of LFchimera plus antibiotics in *Vibrio* resistant strains could be due to the damage exerted by bLF and LFchimera on outer membrane of *Vibrio* spp plus the effect of the antibiotics.

The marked effect of LFchimera could be explained by its composition. LFchimera is formed by the peptides LFcin17-30 and LFampin265-284 (linked by a lysine). In consequence, this new peptide presents the following characteristics; an artificial conformation that mimics the spatial arrangement of the LF native, and a net charge of 12+ at neutral pH (compared with 6+ from LFcin17-30 and 4+ from LFampin265-84; respectively) (Bolscher et al., 2009). In this sense, it has been reported that the negatively charged membrane molecules present in pathogens are the main target of cationic antimicrobial peptides, so we speculate that Fchimera can act with the negatively charged microbial membrane components and destabilizes it, causing antimicrobial effect. Additionally, it has been reported that the bactericidal effect of LFchimera was not hampered by salt concentrations present in the media of bacterial growth.

Until the best of our knowledge this is the first report of the bactericidal activity of bLF and synthetic LFpeptides (LFin17-30, LFampin265-285, and LFchimera) on *Vibrio* species resistant to antibiotics.

In this study, we sought to get further insight into this mechanism by focusing our studies on *V. cholerae* O1 and non-O1 strains due to *V. cholera* is the most pathogenic specie of genus *Vibrio*, for this reason we made experiments to assess the mechanism of action. We found that bLF and LFpeptides caused membrane perturbation in both *V. cholera* strains (Figure 2) as well as damage on structural level (Figures 3, 4, respectively). Certainly, the measurement of FITC-labeling peptides by flow cytometry and microscopy indicated interaction of them with the bacteria (Figure 5), this interaction could then permits the damage of *V. cholerae* membranes. The interaction was visible by confocal microscopy (Figure 5) and was quantified by flow cytometry (data not shown). In additional experiments, we pre-incubated bacteria with high amounts of unlabeled bLF and then the FICT-peptides were added. In all cases bLF did not avoid the binding of peptides to the outer membrane of *V. cholera*, however we found a significant decrease in the fluorescent exhibited by bacteria, indicating that bLF and LFpeptides uses the same sites of recognition present in *V. cholerae*, and maybe specific sites (data not shown). Together this data shown that *V. cholera* contains sites on its membrane that bind bLF and LF peptides.

It has been reported that bLF interacts in a direct manner with negative charged components present in microbial membranes; inducing alterations in its permeability through dispersion of them. For example; bLF interacts with lipopolysaccharides (LPS) from Gram-negative bacteria, or with Lipoteichoic acid (LTA) from Gram-positive bacteria. After this interaction it has been postulated that there is an alteration on membranes, leading the death of the pathogens (Ellison et al., 1988; Orsi, 2004; Leon-Sicairos et al., 2014). We speculate that lipid A from LPS is also one of the targets form LFchimera and LFpeptides. In our precious work LFchimera produced damage on *V. parahaemolyticus*, the appearance of the bacteria shown typical perturbations of a bacteria undergoing programmed cell death type II (Leon-Sicairos et al., 2009), as these kind of damage was not found in all *Vibrio* tested we think LFchimera exerts different type of damage.

Treatment for *V. cholerae* infection involves antibiotics and oral hydration for cholera since 1964. Hydration includes the drinking of fluid with electrolytes, such as sodium, potassium, calcium ions to restore the high amount of electrolytes lost due watery diarrhea (Seas et al., 1996). Regarding drugs, tetracycline has been and effective treatment for cholera with better effect compared with others antibiotics such as furazolidone, chloramphenicol, and sulfaguanidine in reducing cholera morbidity (Lewis and Sanyal, 1965; Gharagozloo et al., 1970; Finkelstein, 1996; Escobar et al., 2015). However, it has been demonstrated the resistance to the antibiotic tetracycline and others (used for *V. cholerae*) in both; endemic and epidemic cholera settings.

Concerning our model *in vivo*, in was clear that LFchimera and bLF were effective to resolve *V. cholerae* infection in mice. LFchimera and bLF had bactericidal activity against the bacteria, and this was confirmed by the resolution of macroscopic damage (Figures 6D,E) and by the diminution of *V. cholerae* counts in feces and intestines of mice infected and treated, compared whit those infected and untreated. It seems to be that the bactericidal effect of LFchimera and bLF was better in comparison with tetracycline. So, In our model LFchimera and bLF were effective against *V. cholerae* infection.

Antibiotic resistance can be acquired by the acquisition of selected mutations, plasmids, introns, or conjugative elements, which could confer rapid spread of resistance (Towner et al., 1980; Hassan and Teh, 1993; Weber et al., 1994; Bhattacharya et al., 2011). Furthermore, it has been demonstrated that in cholera; the mass supply of antibiotics for prophylaxis in asymptomatic persons and household contacts of cholera patients during previous epidemics, represented a risk factor for the acquisition of resistance of *V. cholera* to antibiotics employed (Kitaoka et al., 2011; Marin et al., 2014). Treatment of infections due to *Vibrio* non cholerae also has been difficult in recent times, due to the spread of multidrug resistant *Vibrio* spp strains.

These facts indicated that is necessary searching for new products and interventions that can combat *V. cholerae* and other *Vibrio* spp., because of the increasing resistance against antibiotics. LFchimera and bLF at low concentrations were antibacterial against *Vibrio* spp.; we speculate that both compounds present potential to prevent or combat infections caused by *Vibrio* spp. On the other hand, antibiotics combined with LFchimera could act together, this also represent potential of new option against infections caused by the multidrug resistant *Vibrio* species. In addition, LFchimera could be used as an antibacterial in seafood or in humans, but first its efficacy as food preservative and in *in vivo* must to be determined.

## Conclusions

We performed a study in order to see if bLF and LFpeptides (LFcin17-30, LFampin 265-284, and LFchimera) are effective as bactericides in *V. cholera* O1 and non-O1 strains and other *Vibrio* spp resistant to antibiotics. Data reported here demonstrated that LFchimera and the native bLF are bactericide peptides that damage *Vibrio* spp after a direct interaction. On the other hand, LFchimera and bLF combined with antibiotics could have a combinatory effect against *Vibrio* spp., for this reason they have potential as bactericidal agents against infections caused by *Vibrio* spp.

## Ethics statement

Mice were purchased and housed under specific-pathogen-free conditions, treated and finally killed as stipulated and approved by the Ethical Committee for Laboratory Animals in School of Medicine, University of Sinaloa.

## Author contributions

EA-S, KV-J, AC-R, MR-L, JB, KN, HF-V, GA-C, MdlG, JM-G, and JV-R: Substantial contributions to the conception of the work; acquisition of data and analysis; Drafting the work; Final approval of the version to be published. Agreement to be accountable for all aspects of the work in ensuring that questions related to the accuracy or integrity of any part of the work are appropriately investigated and resolved. NL-S: designed the work; analyzed and interpreted the data for the work; Revised the work for important intellectual; Final approval of the version. She is in Agreement to be accountable for all aspects of the work in ensuring that questions related to the accuracy or integrity of any part of the work are appropriately investigated and resolved.

### Conflict of interest statement

The authors declare that the research was conducted in the absence of any commercial or financial relationships that could be construed as a potential conflict of interest.
